# Feline Morbillivirus in Southern Italy: Epidemiology, Clinico-Pathological Features and Phylogenetic Analysis in Cats

**DOI:** 10.3390/v13081449

**Published:** 2021-07-25

**Authors:** Giulia Donato, Marisa Masucci, Eliana De Luca, Angela Alibrandi, Massimo De Majo, Shadia Berjaoui, Camillo Martino, Cyndi Mangano, Alessio Lorusso, Maria Grazia Pennisi

**Affiliations:** 1 Department of Veterinary Sciences, University of Messina, 98168 Messina, Italy; marisa.masucci@unime.it (M.M.); mdemajo@unime.it (M.D.M.); cyndi_m@hotmail.it (C.M.); mariagrazia.pennisi@unime.it (M.G.P.); 2 Istituto Zooprofilattico Sperimentale dell’Abruzzo e Molise (IZSAM), 64100 Teramo, Italy; elianadeluca9@gmail.com (E.D.L.); s.berjaoui@izs.it (S.B.); c.martino@izs.it (C.M.); a.lorusso@izs.it (A.L.); 3 Department of Economy, University of Messina, 98168 Messina, Italy; angela.alibrandi@unime.it

**Keywords:** cat, feline morbillivirus, FeMV, paramyxovirus, PCR, IIF, epidemiology, chronic kidney disease, CKD

## Abstract

Feline morbillivirus (FeMV) was isolated for the first time in 2012 with an association with chronic kidney disease (CKD) suggested. This study aimed at investigating in cats from southern Italy FeMV prevalence and risk factors for exposure to FeMV, including the relationship with CKD; sequencing amplicons and analyzing phylogeny of PCR positive samples. Blood serum, K_3_EDTA blood and urine samples from 223 cats were investigated. Ten carcasses were also evaluated. FeMV RNA was detected in 2.4% (5/211) blood and 16.1% (36/223) urine samples. One carcass tested positive by qPCR_FeMV_ from kidney, urinary bladder, and submandibular lymph nodes. Antibodies against FeMV were detected in 14.5% (28/193) cats. We followed up 27 cats (13 FeMV positive cats) and documented in some cases urine shedding after up to 360 days. Older and foundling cats and cats living in rescue catteries, were more frequently infected with FeMV. A significant correlation between FeMV and higher serum creatinine values or low urine specific gravity was found. FeMV positivity was significantly associated with retroviral infection, and the presence of some clinical signs apart from CKD clinicopathological markers. Our study highlights the possibility of a link between FeMV exposure and CKD and a general impairment of feline health.

## 1. Introduction

The *Morbillivirus* genus, family *Paramyxoviridae*, includes a group of enveloped viruses of humans and animals with a non-segmented, negative strand RNA genome [1]. In 2009, feline morbillivirus (FeMV) was isolated for the first time from stray cats (*Felis silvestris catus*) in Hong Kong [1,2,3,4]. After the first isolation, FeMV has been worldwide detected in other countries from Far East as Japan, Thailand, Malaysia, mainland China but also in Turkey, Italy, Germany, UK, USA, Chile and Brazil [2,5,6,7,8,9,10,11,12,13,14,15,16,17,18,19,20,21,22,23]. Molecular prevalence reported ranged between 2.7% and 50.8% in urine [2,3,6,8,9,12,13,14,16,18,20,24,25,26], between nihil and 10% in blood [3,8,26], between 2.7% and 80% in kidney tissues [2,6,8,16,20,26], while seroprevalence was between 18.9% and 66.7% [2,3,18,26]. Since the discovery, an association between FeMV infection and tubulointerstitial nephritis (TIN) and glomeruolosclerosis has been suggested [2,3,15,16,19]. TIN and renal fibrosis are the end-stage histological patterns associated with chronic kidney disease (CKD) caused by different causative agents [27]. CKD is a common feline disease with higher morbility and mortality in older individuals, but the underlying cause of CKD remains often unknown [27,28,29]. Before FeMV discovery, viral agents considered potentially involved in feline CKD were feline immunodeficiency virus (FIV), feline leukemia virus (FeLV) and feline coronavirus (FCoV) [27,30]. Despite the positive association between FeMV positivity and CKD detected in some studies [12], currently this relationship remains controversial and not found in some others [6,11,14,16,18,20,26,31,32].

This study primarily aimed at investigating, in cats from southern Italy, the FeMV prevalence and risk factors for exposure to FeMV, including the relationship with CKD; sequencing amplicons and analyzing phylogeny of PCR positive samples.

## 2. Materials and Methods

### 2.1. Power and Sample Size

Sample size calculation for cat enrollment was based on the primary outcome, which is the molecular prevalence of FeMV in Italy. We assumed for this study a known population prevalence of 23.3%, according to previous studies [11,15], and an expected sample prevalence of 15.5%. We assumed a power of 80% and a 2-sided significance level of 5%, and the sample size calculation suggested that the minimum number of cats, equal to 211, was necessary to guarantee an adequate power of study.

### 2.2. Study Sites, Cat Enrollment and Sampling Procedures

Between April 2017 and May 2019, cats aged ≥ 6 months were enrolled at four Veterinary Clinics located in Sicily (Ospedale Veterinario Universitario Didattico, Università degli Studi di Messina, Messina; Ambulatorio Veterinario S. Lucia, Lipari-Messina) and Calabria (Clinica Veterinaria Camagna, Reggio Calabria; Ambulatorio Dr. Cardone, Gioia Tauro-Reggio Calabria). Signalment, clinical history and physical examination findings were registered in a clinical form. Data recorded are listed in Table 1. Body condition score (BCS) was assessed as follows by using a five-point body condition scoring system: 1 = very thin, 2 = thin, 3 = ideal, 4 = overweight, 5 = obese [33]. Muscle condition score (MCS) was assessed using a four-point scoring system according to WSAVA guidelines [34]: 1 = normal, 2 = mild loss, 3 = moderate loss, 4 = severe loss.

Three to five milliliters of blood were taken from each cat: one milliliter was placed into a K_3_EDTA tube, used within 24 h for complete blood count (CBC) and stored at +4 °C until use for detection of FeMV RNA. Remaining blood was used to make blood smears and to obtain serum after clotting in a dry tube and centrifugation. Blood serum was stored at −20 °C until further use for biochemical and serological investigations. Urine samples (about five ml) were obtained by cystocentesis or free catch and used for urinalysis within two hours after collection. The supernatant was used for the evaluation of urine protein to creatinine ratio (UPC) within 24 h after collection. An aliquot of each urine sample was stored at +4 °C and used for detection of FeMV RNA. Similarly, 111 tissues samples (brain, heart, lung, intestine, liver, pancreas, kidney, bladder, spleen, iliac, mesenteric, popliteal, prescapular, submandibular lymph nodes) collected from 10 carcasses, admitted at University of Messina for necropsy, were included in the molecular investigation.

When possible, cats were additionally assessed following the same clinical procedures. The CBC, serum biochemistry and urinalysis have also been re-evaluated whenever possible.

### 2.3. Clinicopathological Evaluation

The CBC was performed using a laser haematology analyzer (IDEXX ProCyte Dx^®^ Hematology Analyzer, Idexx Laboratories, Westbrook, ME, USA). Blood smears were stained by May-Grünwald-Giemsa staining and examined to confirm thrombocytopenia and leukocytes differential count [35]. Values of hemoglobin, neutrophils, lymphocytes, monocytes, eosinophils, basophils, thrombocytes were considered outliers when outside the reference range of more than 10%. A renal profile including blood urea nitrogen (BUN), serum creatinine (sCr), symmetric dimethylarginine (SDMA) and phosphorus (PHOS) was performed by Catalyst Dx^®^ Chemistry Analyzer (BUN, sCr and PHOS) (Idexx Laboratories, Westbrook, ME, USA) and with liquid chromatography-mass spectrometry (SDMA) (IDEXX Laboratories, Novara, Italia S.r.l). When enough amount of serum was available, total thyroxine (tT4) was evaluated in senior cats, at Biogene laboratory (Catania, Italy) with an enzyme linked fluorescent assay (ELFA). Urinalysis was performed by dipstick analysis (Combur 9 Test strips, Roche Diagnostics, Indianapolis, IN, USA), urine specific gravity (USG) was measured by Vet 360 refractometer (Reichert, Seefeld, Germany) and microscopic evaluation of urine sediment by using the Kova glasstic slides (Kova International, Garden Grove, CA, USA). The UPC was assessed with Catalyst Dx^®^ Chemistry Analyzer (Idexx Laboratories, Westbrook, ME, USA), using 0.4 as cut-off value for proteinuria; UPC values between ≥0.2 and ≤0.4 were considered as borderline proteinuria [36]. Reference intervals for the CBC parameters statistically evaluated, tT4 and the renal profile parameters are listed in Table 1.

### 2.4. Clinical Groups

Cats were assigned to a clinical group according to history, physical examination, USG, UPC, sCr, SDMA values and additional laboratory data available. Cats with sCr > 2.4 mg/dL and/or SDMA > 14 µg/dL and/or USG < 1035 and/or UPC > 0.4 were considered affected by CKD (CKD group) [36]. Cats with none of the above abnormalities were considered not affected by CKD and based on the absence of physical examination and clinicopathological abnormalities were classified as “healthy”. The “unhealthy without CKD” group included cats with physical examination and clinicopathological abnormalities different from the CKD. Conversely, cats of the CKD group could also be affected by other physical examination and clinicopathological abnormalities along with CKD. Cats were not assigned to any group (*n* = 31) when recent or current IV fluid treatment at the time of sampling was reported, urinalysis, sCr and/or SDMA evaluations were missing, or pyuria, bacteriuria, and hematuria were detected by urinalysis.

### 2.5. Molecular Detection of FeMV RNA and Other Relevant Feline Pathogens in Cats

Nucleic acids were purified from EDTA-blood samples using the High Pure Viral Nucleic Acid Kit (Roche Life Science, Roche Diagnostics, Monza, Italy) and from urine and tissue using the QiAamp Viral RNA Mini kit (Qiagen, Hilden, Germany). All samples were tested by a quantitative RT-PCR targeting a 76-bp region of the P/V/C gene for detection of FeMV RNA (qPCR_FeMV_) previously developed by the study group at IZSAM (Istituto Zooprofilattico Sperimentale dell’Abruzzo e del Molise) [37]. Primer sequences were 5′- GGGATCCAGAGGGTAACCT -3′ (FeMVrt-F), and 5′-CCGGCCATTAATCTCTGAA -3′ (FeMVrt-R), while the FeMVrt TaqMan probe sequence was FAM-5′-TATTCGAAAGCGATGATGATGAAAACCATTA-3′-TAMRA. The reactions was optimized using the Quantitect Probe RT-PCR Kit (Qiagen, Hilden, Germany) as follows: the 25 μL reaction volume contained 5 μL of purified RNA, 12.5 μL of 2× QuantiTect Probe RT-PCR Master Mix, 0.25 μL of QuantiTect RT Mix, 1.5 μL of each primers (final con-centration 600 nM), 0.625 μL of probe (final concentration of 250 nM), and nuclease-free water up to final volume. The assay was carried out on a 7900HT Fast Real Time System cycler (Applied Biosystem, Waltham, MA, USA) using the following thermal profile: 1 cycle of reverse tran-scription at 50 °C for 30 min, 1 cycle of PCR initial activation step at 95 °C for 15 min followed by 45 cycles of 94 °C for 30 s and 55 °C for 1 min. EDTA-blood samples and tissues of carcasses were tested for feline coronavirus (FCoV) (Dual IPC-TaqVetTM, LSI, Lissieu, France), feline immunodeficiency virus (FIV) (gag protein gene-genesig^®^ Advanced Kit, Rownhams, UK) and feline leukemia virus (FeLV) (U3 region LTR- genesig^®^ Advanced Kit, Rownhams, UK). DNAs were tested for feline parvovirus (FPV) as previously described [38].

### 2.6. Indirect Immunofluorescence Assay for Anti-FeMV Antibodies and Enzyme-Linked Immunosorbent Assay for Anti-FIV Antibodies Detections

The presence of anti-FeMV antibodies (Abs) in serum samples was evaluated by indirect immunofluorescence (IIF) following the procedure recently described by the study group at IZSAM [26]. In addition, serum samples were tested in enzyme-linked immunosorbent assay (ELISA) for the presence of anti-FIV antibodies (Abs, Pet Chek FIV Antibody test kit; IDEXX Laboratory, Westbrook, ME, USA).

### 2.7. Histopathological Examination

Histopathological examination was performed by the “Histology section” of IZSAM. Hematoxylin and eosin staining (HE) and immunohistochemistry (IHC) were performed on formalin fixed paraffin embedded kidney tissue of qPCR_FeMV_ positive carcasses following the procedure recently described by the study group at IZSAM [26].

### 2.8. Sequencing and Phylogenetic Characterization

All positive samples by qPCR_FeMV_ were also tested by a RT-PCR targeting a 401-bp portion of the L protein encoding gene sequence of FeMV with primers FeMV fwd 5′- AAGTATCCTTCAAACACCGAGT -3′, and FeMV rev 5′- TTGAGTAACTCCAAGATGAGGG- 3′, designed by the study group at IZSAM by multiple alignments of the FeMV L encoding gene sequences available online [26]. The reaction was optimized using the QIAGEN OneStep RT-PCR kit (Qiagen, Hilden, Germany) as it follows: the 25 μL of reaction contained 5 μL of 5× QIAGEN OneStep RT-PCR Buffer, 1 μL of dNTPs Mix (10 mM each), 1 μL of QIAGEN OneStep RT-PCR Enzyme Mix, 1.5 μL of each primer (10 μM each), 10 μL of RNAase free water and 5 μL of purified RNA. cDNA was synthesized at 48 °C for 50 min with denaturation at 95 °C for 15 min. The amplification reaction was carried out for 40 cycles with denaturation at 94 °C for 30 s, annealing at 54 °C for 30 s and elongation at 72 °C for 1 min. Resulting amplicons were purified using the QIAquick PCR Purification kit (Qiagen, Hilden, Germany) and sequenced by the Big Dye Terminator v.3.1 kit (Applied Biosystems, Waltham, MA, USA) in the 3130 XL Genetic Analyzer (Applied Biosystems, Waltham, MA, USA) with both primers. All homologous publicly available FeMV sequences were recruited from GenBank and the alignment was performed using the Multiple Alignment using Fast Fourier Transform (MAFFT) software [39]. The phylogenetic analysis was inferred using the Maximum Likelihood (ML) method based on the Tamura 3-parameter model [40]. The evolutionary analysis was conducted in MEGA 7.0 [41].

### 2.9. Statistical Analysis

Descriptive statistic was performed for all the evaluated numerical variables. A non-parametric approach was used since almost all variables were not normally distributed, as verified by the Kolmogorov-Smirnov test.

Spearman’s Rho test was used to measure the strength of correlation between the investigated variables. Therefore, correlations between FeMV urine (u^+^), blood (b^+^), urine and/or blood (u^+^ and/or b^+^) positivity by qPCR_FeMV_, FeMV Abs (Ab^+^), FeMV Abs and/or RNA (Ab^+^ and/or RNA^+^) (urine and/or blood) detection and age group, breed and other 22 variables reported in Table 1 (except for the tT4) were investigated. Fisher’s exact test was used to evaluate differences related to signalment data of the enrolled cats between Sicily and Calabria and the relationship between FeMV detection (u^+^, b^+^, u^+^ and/or b^+^, Ab^+^, Ab^+^ and/or RNA^+^) and cat age. Pearson’s chi-squared test or likelihood-ratio test was used to evaluate the relationship between FeMV detection (u^+^, b^+^, u^+^ and/or b^+^, Ab^+^, Ab^+^ and/or RNA^+^) and other categorical variables such as sex, clinical status, environment, lifestyle, type of indoor lifestyle (multi-cat household, single-cat household, rescue cattery) and origin of indoor cats (foundling, not-foundling) and the association between FeMV and the other investigated pathogens.

Fisher’s exact test was used to evaluate the relationship between the four infection patterns obtained with molecular (qPCR_FeMV_ u and/or b) and serological (Ab) investigations related to FeMV and the investigated variables reported in Table 1 and the other viruses evaluated.

To identify the main risk factors for FeMV positivity and its role on the development of CKD when compared with other infectious pathogens, three multivariable logistic regression analysis models (Stepwise logistic regression analysis) were developed. The first model included five covariates (sex, age, breed, lifestyle, and environment) considered as possible risk factors for urine and/or blood qPCR_FeMV_ positivity; the second model included the same five variables considered as possible risk factors for FeMV Abs positivity; the third model included five variables (urine and/or blood qPCR_FeMV_ positivity, FeMV Abs positivity, FIV antibody and/or PCR positivity, FCoV PCR positivity, FPV PCR positivity) considered as possible predictors of CKD development. Statistical analysis was performed using SPSS 22.0 for Windows package. *p*-values lower than 0.05 were considered statistically significant.

## 3. Results

### 3.1. Cat Population Demographic and Clinical Data

Two hundred and twenty-three cats were enrolled, with 74 cats from Sicily and 149 from Calabria regions, respectively. From the 223 enrolled cats: 211 K_3_EDTA blood samples, 223 urine samples and 193 blood serum samples were collected. Signalment and history of the enrolled cats are shown in Table 2. Cats were aged between 5 and 216 months (median 36 months, 25th percentile 12 months, 75th percentile 96 months). Cats coming from Sicily and Calabria differed in age, breed, lifestyle, and environment. Particularly, adult (Fisher’s exact test, *p* = 0.0399) and senior (Fisher’s exact test, *p* = 0.004) cats as well as pure-breed cats (Fisher’s exact test, *p* = 0.002) were more frequently enrolled in Sicily and most of the Sicilian cats were from rescue-catteries (Fisher’s exact test, *p* = 0.0080) and from suburban (Fisher’s exact test, *p* = 0.0001) and rural areas (Fisher’s exact test, *p* = 0.001). Based on the above differences, prevalences of FeMV in the two regions were not compared.

Total T4 was evaluated in 38 of the 53 senior cats. Ten of the tested cats (26%) had high tT4 values, included four cats with CKD and two of them were FeMV positive.

Clinical findings of the enrolled cats are shown in Table 3. In detail, upper respiratory tract signs reported were nasal discharge, sneezing, and stertor; dyspnea was the lower respiratory tract sign observed; gastrointestinal signs included vomiting, diarrhea and abdominal pain; skin lesions were alopecia, ulcers, crusts, nodules, cysts, and comedones; conjunctivitis, keratoconjunctivitis, and keratouveitis were seen at eye examination; stomatitis was the lesion observed in the oral cavity. Prevalence of CBC and renal profile abnormalities in enrolled cats are shown in Table 4. According to clinical and clinicopathological evaluations, 88 (45.8%), 92 (47.9%) and 12 (6.3%) enrolled cats were included in the “CKD”, “unhealthy without CKD”, and “healthy” clinical groups, respectively.

### 3.2. FeMV RNA and Anti-FeMV Antibodies Were Detected

The 2.4% of blood samples (5/211 cats) and the 16.1% of urine samples (36/223 cats) were qPCR_FeMV_ positive, with an overall molecular prevalence of 18.5%. Two cats were positive from both urine and blood samples, 33 cats resulted qPCR_FeMV_ positive only from urine whereas three cats were only qPCR_FeMV_ positive from blood. Abs against FeMV were detected in the 14.5% (28/193 cats) of samples tested. Fifty-six cats out 184 were positive to at least one test with an overall prevalence (cats antibody and/or PCR positive) of 30.4% (Table 2).

The 10% of the carcasses (1/10 cats) tested positive by qPCR_FeMV_ from kidney, urinary bladder, and submandibular lymph nodes. The positive carcass originated from a cat which was demonstrated to shed FeMV RNA also *intra vitam*.

### 3.3. FeMV Detection Was Related with Some Investigated Variables

FeMV detection was positively related to age (“u^+^ and/or b^+^” *r_s_* = 0.151, *p* = 0.031; “Ab^+^ and/or RNA^+^” *r_s_* = 0.165, *p* = 0.025) and FeMV Ab^+^ and/or RNA^+^ prevalence was higher in senior (Fisher’s exact test, *p* = 0.0378) cats compared to the junior cats. Additionally, cats from single-cat household (Chi-squared test, “u^+^” Χ^2^ = 6.9, degrees of freedom (dof) = 2, *n* = 108, *p* = 0.032; “u^+^ and/or b^+^” Χ^2^ = 6.9, dof = 2, *n* = 98, *p* = 0.033) and rescue catteries (Chi-squared test, “Ab^+^” Χ^2^ = 10.1, dof = 2, *n* = 90, *p* = 0.006; “Ab^+^ and/or RNA^+^” Χ^2^ = 8.1, dof = 2, *n* = 87, *p* = 0.018) were more frequently found positive compared to multi-cat household as well as foundling cats (Chi-squared test, “Ab^+^” Χ^2^ = 4, dof = 1, *n* = 90, *p* = 0.046) compared to not foundling cats (Table 2).

Clinical findings of FeMV positive cats are shown in Table 3. FeMV exposure (Ab^+^) was negatively related to BCS and positively related to gastrointestinal signs, oral and skin lesions. FeMV detection (u^+^, b^+^, u^+^ and/or b^+^) was positively related to upper and lower respiratory tract signs, gastrointestinal signs and ocular lesions (Table 3). The CBC changes correlated with FeMV exposure and/or detection were anemia, neutrophilia, basophilia, eosinopenia, monocytosis, and thrombocytosis (Table 5). Significant renal profile changes in FeMV positive cats included increased sCr (Ab^+^) values and low urine specific gravity (b^+^) at urinalysis (Table 5).

The prevalence of FeMV positive cats in the three clinical groups is represented in Table 6, however no significant differences were found.

### 3.4. FeMV Infection Was Associated with Retroviral Infections

Prevalence of the other investigated viruses is reported in Table 7. Significant associations were found between u^+^ and/or b^+^ qPCR_FeMV_ and positivity to FIV (Chi-squared test, Χ^2^ = 8.7, dof = 1, *n* = 200, *p* = 0.003), as well as with PCR positivity to FeLV (Chi-squared test, Χ^2^ = 4.7, dof = 1, *n* = 205, *p* = 0.031) and between PCR positivity to FCoV and FPV (Likelihood ratio, *p* = 0.029). Significant correlations between FeMV and FIV and FeMV and FCoV coinfections and the investigated variables are reported in Table 8. No significant correlations were found between FeMV and FeLV coinfection and the investigated variables.

### 3.5. FeMV Patterns Based on Molecular and Serological Data Were Associated with Some Investigated Variables

Based on molecular and serological data, four different patterns were evidenced, and they are reported in Table 9.

Significant associations found between the four infection patterns and the investigated variables are reported in Table 10.

### 3.6. Older Age Was a Risk Factor for FeMV Infection and Seropositivity to FeMV Was Associated with CKD

The first multivariable logistic regression model found older age (*p* = 0.019, OR= 1.708, 95% CI = 1.091–2.675) as possible risk factor for u^+^ and/or b^+^ qPCR_FeMV_. The second model did not show possible risk factors for FeMV Ab^+^ among the evaluated variables. The third model found FeMV Ab^+^ (*p* = 0.049, OR = 3.097, 95% CI = 1.004–9.550) was significantly associated to CKD among the five viral pathogens investigated.

### 3.7. Immunoreactivity to FeMV Was Observed in Renal Tubules

**Figure 1:**
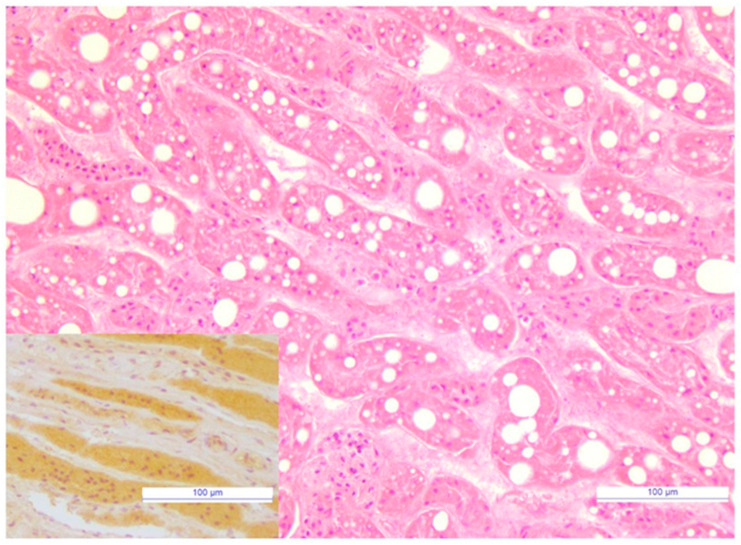
Cat, Kidney. Diffuse lipidosis of cortical tubular epithelial cells. H&E, Bar = 100 μm. *Inset:* Cat, Kidney. Tubular structures of medulla showed strong and diffuse immunoreactivity for FeMV. IHC, Mayer’shaematoxylincounterstain, Bar = 100 μm.

Histopathological examination and IHC were performed on the renal tissue of a unique carcass tested positive by qPCR_FeMV_. The cortical epithelial cells of proximal tubules were markedly dilated by variably-sized clear cytoplasmic lipid vacuoles (Figure 1). Intraluminal minerals were also observed in some proximal tubules. Strong and diffuse intracytoplasmic immunoreactivity for FeMV was observed in proximal tubular structures of renal cortex as well as in non-degenerate tubular structures of medulla (Figure 1).

### 3.8. FeMV Genotype 1 Was Sequenced

**Figure 2:**
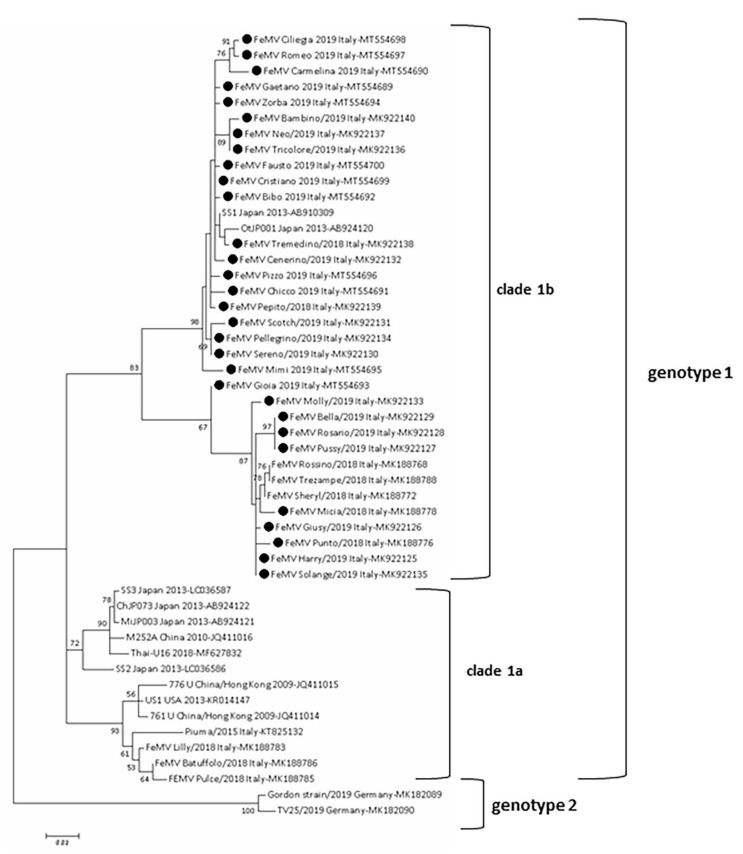
Phylogenetic analysis based on a 400-bp L gene nucleotide sequences of 27 FeMV strains of this study and FeMV sequences publicly available. The analysis involved a total number of 50 nucleotide sequences. The tree with the highest log likelihood (−1706.64) is shown. The percentage of trees in which the associated taxa clustered together is shown next to the branches. Initial tree(s) for the heuristic search were obtained automatically by applying Neighbor-Join and BioNJ algorithms to a matrix of pairwise distances estimated using the Maximum Composite Likelihood (MCL) approach, and then selecting the topology with superior log likelihood value. A discrete Gamma distribution was used to model evolutionary rate differences among sites (5 categories (+G, parameter = 0.3706). The tree is drawn to scale, with branch lengths measured in the number of substitutions per site. There was a total of 370 positions in the final dataset. Evolutionary analyses were conducted in MEGA7. Black circle: FeMV sequences obtained in this study.

A total of twenty-seven L gene sequences was obtained from twenty-six urine samples and one blood sample, respectively. All sequences belonged to FeMV genotype 1 (Figure 2). FeMV sequences cluster in two different clades (clade 1a and clade 1b) within genotype 1, in accordance with what observed in a previous study of the IZSAM group [26]. The FeMV sequences of the current study cluster in the clade 1b, and they share the highest nt sequence identity with the Japanese strains OtJP001 and SS1 (97.3–98.9 and 99.7–98.1%, respectively), whereas the lowest nt identity was evidenced with early FeMV strains 761U and 776U isolated in China in 2009 (89.2–90.3% and 89.2–90.3%, respectively) [3]. On the other hand, sequences belonging to clade 1a shared an identity of 88.6–92.7% with publicly available sequences, including those from this study.

### 3.9. Data about FeMV Viremia, Urinary Shedding, Seroreactivity and CKD in Followed Up Cats

Twenty-seven cats were additionally assessed over a 7–570 days period (Table 11): 14 cats were always found negative and 13 cats were positive at one or more evaluations. Four of the 13 positive followed up cats were affected by CKD since their enrollment in the study (cats 2, 7, 9 and 10), one cat was not staged for the lack of USG values (cat 4), seven cats did not show CKD abnormalities during the follow up (cat 1, 3, 5, 6, 8, 12, 13). Only one cat (cat 11) had an increase of SDMA and sCr values, and a reduction of USG values at the end of the follow up (day 510).

## 4. Discussion

In this study we identified the presence of FeMV in cats from Sicily and Calabria (Southern Italy), in line with previous studies conducted in Italy [10,20,26,37,42,43]. A prevalence of 2.4% in blood, 16.1% in urine samples and a high percentage of cats with detectable serum Abs against FeMV (14.5%) were observed. The 30.4% overall prevalence (cats antibody and/or PCR positive) shows that the circulation of the virus involved about one third of the population under study. These results are similar to those recently reported by *De Luca* and others who evaluated FeMV prevalence in cats from Teramo (Abruzzo region) and Bologna (Emilia-Romagna region) [26], but higher than those reported by *Stranieri* and others in cats from Milan (Lombardy region) [20]. These differences could be related to the demographic characteristics of cats enrolled, as well as to the different PCR assays used. Indeed, a significant association between FeMV positivity and foundling origin of cats or rescue cattery environment was found in this study, consistent with the positive association between FeMV infection and outdoor access reported by *Yilmaz* and others [16]. On the contrary, most of the cats enrolled in the study described by *Stranieri* and others [20], were client-owned, and this could account for the discrepancy in the FeMV prevalence values observed. Moreover, we used a quantitative PCR assay previously developed by the study group at IZSAM [37], which has a higher sensitivity than the conventional RT-PCR assay used by *Stranieri* and others [20,32].

There are still few data about potential risk factors for FeMV exposure, with male sex, young age and multi-cat environments associated with high FeMV positivity rates [2,6,14,23,26,44]. In contrast with the findings of *Busch* and others [44], we found that older age was associated with FeMV positivity, supporting the hypothesis of a chronic course of the viral infection [10,13,29]. Differently from previous studies [14,26], we found that single-cat household cats were more frequently positive than multi-cat household. However, these cats have all been adopted in late age before their enrollment in the study and FeMV infection may have occurred before their adoption.

As previously reported [2], four different patterns resulted from the possible combinations of molecular and serological data in cats tested in this study. These patterns were interpreted as possible markers of the phase of FeMV infection [2,29]. However, cross sectional evaluations have predictable limitations for this aim. To overcome them, we tried to follow up as many cats as possible according to owner compliance, and thus, frequency and duration of monitoring with urine and/or blood samplings were variable. Almost 70% of tested cats were RNA and Abs negative: they have probably never been exposed to FeMV or cleared the infection and converted to an antibody negative status, as we have seen in cat 10. Half of the 56 positive cats were RNA positive and antibody negative: these cats can be considered in the acute early phase of infection, before the development of an immune response. In our study this pattern was associated with the presence of neutrophilia and monocytosis, and both parameters can be related to an inflammatory condition. However, four RNA positive followed up cats (cats 3, 5, 8 and 9) did not seroconvert over their follow up time (21 days–360 days) therefore the interpretation of this pattern requires more extensive information from longitudinal investigations. The RNA and antibody positive cats were a minority of the overall positive individuals (14.3%) and this pattern can represent either an advanced phase of the acute infection or a chronic stage. Interestingly, we found this pattern associated with FIV coinfection and stomatitis. FIV-induced immune-dysfunction could be responsible for a more prolonged course of FeMV infection and stomatitis would be a comorbidity typically described in FIV positive cats [45]. Finally, more than one third of the positive cats were RNA negative and antibody positive and this pattern can be found in different situations. In fact, it can be found in infected individuals that developed an antibody response and eliminated the virus, and *Park* and others proposed that they are in a convalescent phase of infection [2]. However, in the present study this pattern was associated with weight loss, oral lesions and gastrointestinal signs as well as presence of anemia and neutrophilia. Additionally, a discontinuous urinary shedding of viral RNA, as we evidenced for cats 2 and 10, can be missed by a cross-sectional evaluation. We do not know whether reinfections occur or not, however we excluded this possibility for cat 10 that was followed up during a long-term hospitalization. Moreover, we did not perform a daily shedding evaluation, but eight cats were positive in multiple urine samples and up to 360 days after the first detection. We cannot state that in our study the RNA shedding was continuous or whether in some cases there was a reinfection. In fact, two of the positive followed up cats lived in a rescue cattery, four cats were outdoors and only two cats were hospitalized without a direct contact with other cats during the follow up. Similar limitations are found in two previous studies where three FeMV positive cats were followed up and a long-term shedding was reported [13,46].

Interestingly, cat 10 which was followed up for six months, converted to an antibody negative status and it was associated with stopping RNA urinary shedding. This is the first time that conversion to an antibody negative status after stopping RNA urinary shedding is documented in a field study. This finding may suggest a spontaneous clearance of FeMV infection in this cat, as it was already supposed in previous studies [2,29].

As reported before [6,26], we detected FeMV RNA in just five blood samples and only two cats were positive in both urine and blood. This could be due to a short term course of viremia compared to the persistence of urinary shedding [6,12], which may explain the higher prevalence of RNA positivity in urine samples. Additionally, the qPCR_FeMV_ blood positivity along with the lack of qPCR_FeMV_ urine detection before seroconversion in cat 4 may account for this hypothesis. Although viremia is reported to have a shorter duration compared to urinary elimination, two cats (4 and 8) were blood PCR positive in two successive samples, 21–30 days apart.

The pathogenic role of FeMV in CKD is the most important and debated topic, which was the object of most of FeMV studies performed since its discovery [2,3,6,8,9,11,12,14,15,16,18,19,20,26,31,44,47,48,49]. In this study, we did not find different FeMV prevalences between the three clinical groups. However, we did not perform a complete biochemical profile and this limitation could have caused an overestimation of the number of cats classified as “healthy”. We found increased sCr and low USG values in FeMV positive cats, and they are abnormalities suggestive of an impaired kidney function and of a relationship between FeMV positivity and CKD [27]. We evaluated for the first time the role of feline viral pathogens known to be responsible of kidney disease such as FIV and FCoV, using a multivariable logistic regression analysis [27,30]. We found that only FeMV antibody positivity was a possible predictor of CKD. Our findings are in line with the recognized relationship between FeMV infection and kidney diseases (tubulointerstitial nephritis or other renal inflammatory lesions) demonstrated in most of the histopathological investigations and in clinical studies [2,3,8,9,12,16,19,44,47]. Other studies that focused on the relationship between FeMV and azotemic CKD did not support this result [6,11,14,16,18,20,26,31]. The reasons for this discrepancy can be various and many. First of all, FeMV infection can result in a discontinuous RNA urinary shedding, as we found in the followed-up cats, with a possible negative misdiagnosis when a cross sectional evaluation tests a single urine sample per cat [18]. Moreover, many of these studies did not combine RNA detection with anti-FeMV antibody evaluation for assessing the exposure of cats to FeMV. We found that 15.3% of RNA negative cats were antibody positive and their exposure to FeMV would have been missed without testing them with both molecular and serological assays. We have also to underline that CKD develops months or years after the exposure to a pathogen (depending also by co-factors) when the causative agent could have been cleared. In case of FeMV infection, the viral RNA could be no longer detected in urine samples nor anti-FeMV antibodies could be found [18,19]. Furthermore, unlike most previous clinical studies [6,14,16,18,20], we used an early and more sensitive biomarker (SDMA) for the diagnosis of CKD with the possibility to analyze data also from cats with stage-1 CKD [32,50,51]. However, we repeated the assessments of this parameter as well as of sCr and USG values, only in 14 followed-up positive cats. It is noteworthy to point out that only controlled longitudinal studies may clarify whether FeMV acts as a primary pathogen in the CKD development or whether it just benefits from inflamed tissues of the urotract as the basis for a secondary infection [12]. We followed up only 14 positive cats, and 4 of them were affected by CKD at the time of enrollment in the study. Seven cats maintained normal values of SDMA, sCr, USG and UPC during their follow up and just one cat showed worsened values during the 18 months of follow up with development of CKD. However, we did not have a followed-up control group and we cannot exclude that other factors have determined these changes. Indeed, cats included in longitudinal field studies can be exposed to uncontrolled infectious and not infectious CKD risk factors, confounding the analysis of results.

Few studies focused on the association between FeMV positivity and feline clinical conditions other than CKD [16,32,44,49]. *Yilmaz* and others evaluated both clinical, clinicopathological and histopathological abnormalities reporting weight loss, fever, depression, respiratory, urinary, and gastrointestinal signs in FeMV RNA positive cats (3/68 cats), as well as, lower red blood cells, hemoglobin, albumin, albumin/globulin ratio and urobilinogen median values, and higher alanine aminotransferase, alkaline phosphatase and bilirubin median values when compared with negative cats [16]. We evaluated abnormalities related to physical examination, CBC, serum biochemistry and urinalysis in a larger number of positive cats and in accordance with *Yilmaz* and others [16], a low BCS, respiratory and gastrointestinal signs, ocular, oral, and skin lesions were more frequently observed in FeMV positive cats. Moreover, anemia, neutrophilia, basophilia, eosinopenia, monocytosis and thrombocytosis were statistically correlated to FeMV positivity. These CBC abnormalities are consistent with an inflammatory disease, but this latter can be possibly related to comorbidities and coinfections, and this needs to be investigated with a more extensive clinicopathological investigation. Based on in vitro studies, a wide spectrum of FeMV cell tropism can be expected. Indeed, FeMV genotype 1 was able to infect feline epithelial, fibroblastic, lymphoid, and glial cells [9]. Recently, a new genotype of feline morbillivirus, tentatively named feline morbillivirus genotype 2 (FeMV-GT2), was described [46]. This new genotype was able to infect, in vitro, primary feline pulmonary epithelial cells and cells from cerebrum and cerebellum, as well as blood CD4^+^ T cells, and CD20^+^ B cells. These results suggest that there is a diversity of feline paramyxoviruses [46], and that both known genotypes may infect different feline cells [9,26]. Therefore, other tissues than kidney can be involved by FeMV infection and other clinical abnormalities may be expected. In this study, one cat was found qPCR_FeMV_ positive in kidney, urinary bladder, and submandibular lymph nodes samples. However, the HE examination performed on the kidney tissue revealed tubular changes which were not indicative of TIN. Since these lesions are common in cats, it is hard to interpret and discuss their clinical significance. Additional studies with a larger number of carcasses are warranted to establish the relationship between TIN and FeMV infection.

We explored thyroidal function in most of the senior cats as hyperthyroidism, a risk factor for feline CKD. However, we were not able to investigate the clinical status of enrolled cats with a complete biochemical profile, and thus, some clinicopathological abnormalities could have been missed.

Coinfection between FeMV and other viral pathogens have been investigated by few studies. Coinfections with FIV, FCoV, FeLV, and FPV were found in some cats while, recently, *Busch* and others did not find an association between FeMV antibody status and other infectious diseases agents investigated (FIV, FCoV, feline herpesvirus-1, feline calicivirus, *Mycoplasma* sp., FPV, lungworm) [14,20,26,44]. Our study detected a significant association between FeMV and feline retroviral infections. We found that cats positive for both FeMV and FIV were more frequently old cats and they were affected by low BCS and enlargement of lymph nodes more than cats positive only for FeMV. The higher prevalence of FIV is generally related to older age [45], probably as this pathogen is transmitted by aggressive interactions (biting), which typically occurs within adult cats and as the infection lasts for a lifetime. Moreover, emaciation and lymphadenopathy are frequently found in FIV positive cats [45]. This result suggests that coinfection between these two pathogens can worsen the clinical manifestations of FeMV positive cats, or the other way around, and further studies on the role of coinfections, including also other feline pathogens, are advisable.

## 5. Conclusions

Based on our results, feline morbillivirus is widely present in Southern Italy, with older and foundling cats being more frequently infected, as well as cats living in rescue catteries. We found a significant correlation between FeMV exposure and higher sCr values or low USG. Furthermore, FeMV was a predictor of CKD development among the investigated viral pathogens, which are potentially involved in kidney damage, suggesting a potential relationship between FeMV and CKD. Additionally, FeMV positivity was significantly associated with a retroviral infection, and the presence of some clinical signs apart from CKD clinicopathological markers. Finally, FIV coinfection was associated with FeMV molecular positivity in antibody positive cats. Further studies can be useful to assess whether coinfections are responsible for the clinical signs reported in this study or whether they may aggravate the clinical condition of FeMV positive cats and favor prolonged urinary shedding.

## Figures and Tables

**Table 1:** List of variables statistically evaluated (apart from total thyroxine) in the enrolled cats concerning: signalment and history, physical examination findings, complete blood count (CBC) and renal profile parameters with their reference values, and abnormalities considered of clinical interest. *: values ≥ 0.2 ≤ 0.4 were evaluated as border-line proteinuria; ^ foundling cat: stray or abandoned cat adopted months or years before the enrollment in this study.

Signalment and History	Physical Examination	CBC and Renal Profile Parameters [Reference Values]	Abnormality
Sex Male/Female Age group (months) Junior (6–24) Adult (25–96) Senior (>96) Breed Domestic shorthair (DSH) Domestic longhair (DLH) Pure Breed Lifestyle and origin Outoor Indoor Single-cat household Multi-cat household Rescue Cattery Foundling cat ^ Not-foundling cat Environment Urban (area with a high population density) Suburban (sites near outskirts of urban areas) Rural (area with a low population density and small settlements)	Body condition score (BCS) Muscle condition score (MCS) Lymph node enlargement Upper respiratory tract signs Lower respiratory tract signs Gastrointestinal signs Skin lesions Ocular lesions Oral lesions	Hemoglobin (Hb) [8.82–17.82 g/dL] Neutrophils [2.07–11.32 K/µL] Lymphocytes [0.83–7.57 K/µL] Monocytes [0.05–0.74 K/µL] Eosinophils [0.15–1.73 K/µL] Basophils [0.01–0.29 K/µL] Thrombocytes [136–660 K/µL] Symmetric Dimethylarginine (SDMA) [≤ 14 μg/dL] Serum Creatinine (sCr) [0.8–2.4 mg/dL] Blood Urea Nitrogen (BUN) [16–36 mg/dL] Phosphorus (PHOS) [3.1–7.5 mg/dL] Urine Specific Gravity (USG) [>1035] Urine Protein to Creatinine Ratio (UPC) [>0.4] Total Thyroxine (tT4) [1–3 µg/dL]	Anemia Neutrophilia/Neutropenia Lymphocytosis/Lymphopenia Monocytosis Eosinophilia/Eosinopenia Basophilia Thrombocytosis/Thrombocytopenia Increased SDMA Increased sCr Increased BUN/Decreased BUN Increased PHOS USG < 1035 Proteinuria * Increased tT4

**Table 2:** Data from signalment and history [*n* (%)] of enrolled cats and cats positive to FeMV according to their positivity with PCR from urine (u^+^), blood (b^+^), urine and/or blood (u^+^ and/or b^+^), antibody positivity (Ab^+^), antibody and/or qPCR_FeMV_ (Ab^+^ and/or RNA^+^). DSH = domestic shorthair; DLH = domestic longhair.

Signalment and History	Enrolled Cats	u^+^	b^+^	u^+^ and/or b^+^	Ab^+^	Ab^+^ and/or RNA^+^
Sex						
Male	110 (49.3)	21/107 (19.6)	4/106 (3.8)	22/103 (21.4)	16/95 (16.8)	30/92 (32.6)
Female	113 (50.7)	15/110 (13.6)	1/105 (0.9)	16/102 (15.7)	12/98 (12.2)	26/92 (28.3)
Age group						
Junior	91 (40.8)	9/88 (10.22)	2/90 (2.2)	10/87 (11.5)	9/84 (10.7)	18/81 (22.2)
Adult	79 (35.4)	16/77 (20.8)	2/72 (2.8)	16/70 (22.9)	9/64 (14)	21/61 (34.4)
Senior	53 (23.8)	11/52 (21.2)	1/49(2)	12/48 (25)	10/45 (22.2)	17/42 (40.5)
Breed						
DSH	188 (84.3)	33/182 (18.1)	5/179 (2.8)	35/173 (20.2)	25/164 (15.2)	52/156 (33.3)
DLH	23 (10.3)	3/23 (13)	0	3/20 (15)	3/19 (15.8)	4/18 (22.2)
Pure Breed	12 (5.4)	0	0	0	0	0
Maine Coon	9	0	0	0	0	0
Persian	1	0	0	0	0	0
Brithish Shorthair	1	0	0	0	0	0
Ragdoll	1	0	0	0	0	0
Lifestyle and origin						
Outoor	115 (51.6)	21/111 (18.9)	3/111 (2.7)	23/107 (21.5)	19/103 (18.5)	35/97 (36)
Indoor	108 (48.4)	15/106 (14.2)	2/100 (2)	15/98 (15.3)	9/90 (10)	21/87 (24.1)
Single-cat household	32 (29.6)	8/32 (25)	1/28 (3.6)	7/28 (25)	0	7/24 (29.2)
Multi-cat household	35 (32.4)	1/35 (2.9)	0	1/35 (2.9)	1/30 (3.3)	2/30 (6.7)
Rescue Cattery	41 (38)	6/39 (15.4)	1/37 (2.7)	7/35 (20)	8/36 (22.2)	12/33 (36.4)
Foundling cat	57 (52.8)	9/55 (16.4)	1/53 (1.9)	10/51 (19.6)	8/52 (15.4)	15/49 (30.6)
Not-foundling cat	51 (47.2)	6/51 (11.8)	1/47 (2.1)	5/47 (10.6)	1/38 (2.6)	6/38 (15.8)
Environment						
Urban	148 (66.4)	21/145 (14.5)	3/143 (2.1)	21/140 (15)	16/129 (12.4)	33/126 (26.2)
Suburban	61 (27.4)	11/59 (18.7)	2/55 (3.6)	13/53 (24.5)	11/53 (20.8)	18/49 (36.7)
Rural	14 (6.3)	4/13 (30.8)	0	4/12 (33.3)	1/11 (9.1)	5/9 (55.6)
TOTAL	223	36/223 (16.1)	5/211 (2.4)	38/205 (18.5)	28/193 (14.5)	56/184 (30.4)

**Table 3:** Prevalence of clinical findings of enrolled cats and cats positive to FeMV according to their positivity by qPCR_FeMV_ from urine (u^+^), blood (b^+^), urine and/or blood (u^+^ and/or b^+^), FeMV Abs (Ab^+^), FeMV Abs and/or qPCR_FeMV_ (Ab^+^ and/or RNA^+^), with description of Spearman’s rank correlation coefficient (*r_s_*), *p* values and significant difference (*).

Variable	Enrolled Cats	u^+^	b^+^	u^+^ and/or b^+^	Ab^+^	Ab^+^ and/or RNA^+^
Body Condition Score	40/223 (17.9)					
*n* (%)		5/36 (13.9)	0/5	5/38 (13.2)	9/28 (32.1)	13/56 (23.2)
*r_s_*		0.046	0.073	0.055	−0.165	−0.124
*p*		0.492	0.291	0.432	0.022 *	0.094
Muscle Condition Score	50/216 (23.1)					
*n* (%)		8/35 (22.9)	0/5	8/37 (21.6)	8/26 (30.8)	15/53 (28.3)
*r_s_*		0.003	0.086	0.014	−0.083	−0.106
*p*		0.965	0.216	0.840	0.254	0.157
Lymph node enlargement	64/216 (29.6)					
*n* (%)		11/36 (30.6)	0/5	11/38 (28.9)	10/28 (35.7)	17/56 (30.4)
*r_s_*		0.009	−0.103	−0.015	0.041	−0.017
*p*		0.895	0.135	0.831	0.570	0.823
Upper respiratory tract signs	18/216 (8.3)					
*n* (%)		6/36 (16.7)	2/5 (40)	8/38 (21)	4/28 (14.3)	9/56 (16.1)
*r_s_*		0.135	0.183	0.220	0.080	0.173
*p*		0.048 *	0.008 *	0.002 *	0.271	0.019 *
Lower respiratory tract signs	3/216 (1.4)					
*n* (%)		0/36	1/5 (20)	1/38 (2.6)	1/28 (3.6)	1/56 (1.8)
*r_s_*		−0.053	0.244	0.046	0.067	0.008
*p*		0.438	0.000 *	0.512	0.353	0.913
Gastrointestinal signs	19/216 (8.8)					
*n* (%)		6/36 (16.7)	2/5 (40)	6/38 (15.8)	6/28 (21.4)	10/56 (17.9)
*r_s_*		0.124	0.175	0.118	0.183	0.215
*p*		0.068	0.011 *	0.094	0.011 *	0.003 *
Skin lesions	44/216 (20.4)					
*n* (%)		11/36 (30.6)	2/5 (40)	12/38 (31.6)	10/28 (35.7)	17/56 (30.4)
*r_s_*		0.113	0.076	0.130	0.152	0.159
*p*		0.097	0.276	0.064	0.034 *	0.032 *
Ocular lesions	22/216 (10.2)					
*n* (%)		7/36 (19.4)	1/5 (20)	8/38 (21)	4/28 (14.3)	9/56 (16.1)
*r_s_*		0.138	0.052	0.169	0.061	0.125
*p*		0.043 *	0.453	0.015 *	0.396	0.091
Oral lesions	46/216 (21.3)					
*n* (%)		10/36 (27.8)	2/5 (40)	11/38 (28.9)	12/28 (42.9)	18/56 (32.1)
*r_s_*		0.071	0.073	0.099	0.204	0.177
*p*		0.300	0.292	0.159	0.004 *	0.016 *

**Table 4:** Prevalence (%) of complete blood count and renal profile abnormalities in enrolled cats and in cats positive to FeMV according to their positivity with qPCR_FeMV_ from urine (u^+^), blood (b^+^), urine and/or blood (u^+^ and/or b^+^), FeMV Abs (Ab^+^), FeMV Abs and/or qPCR_FeMV_ (Ab^+^ and/or RNA^+^). Hb = hemoglobin; SDMA = symmetric dimethylarginine; sCr = serum creatinine; BUN = blood urea nitrogen; PHOS = phosphorus; USG = urine specific gravity; UPC = urine protein to creatinine ratio; tT4 = total thyroxine; BP = borderline proteinuric; P = proteinuric.

Parameter and Abnormalities	Enrolled Cats	u^+^	b^+^	u^+^ and/or b^+^	Ab^+^	Ab^+^ and/or RNA^+^
**Hb**						
Anemia	41/223 (18.4)	9/36 (25)	1/5 (20)	9/38 (23.7)	9/28 (32.1)	16/56 (28.6)
**Neutrophils**						
Neutrophilia	42/194 (21.6)	11/31 (35.4)	2/5 (40)	11/33 (33.3)	11/28 (39.3)	19/51 (37.2)
Neutropenia	8/194 (4.1)	0/31	0/5	0/33	0/28	0/51
**Lymphocytes**						
Lymphocytosis	6/194 (3.1)	0/31	1/4 (25)	1/33 (3)	0/28	1/51 (1.9)
Lymphopenia	17/194 (8.8)	4/31 (12.9)	1/4 (25)	4/33 (12.1)	1/28 (3.6)	4/51 (7.8)
**Monocytes**						
Monocytosis	34/194 (17.5)	9/31 (29)	1/5 (20)	11/33 (33.3)	7/28 (25)	16/51 (31.4)
**Eosinophils**						
Eosinophilia	10/194 (5.1)	1/31 (3.2)	0/5	1/33 (3)	2/28 (7.1)	2/51 (3.9)
Eosinopenia	20/194 (10.3)	4/31 (12.9)	2/5 (40)	5/33 (15.2)	2/28 (7.1)	6/51 (11.8)
**Basophils**						
Basophilia	1/194 (0.5)	1/31 (3.2)	0/5	1/33 (3)	1/28 (3.6)	1/51 (1.9)
**Thrombocytes**						
Thrombocytosis	7/199 (3.5)	3/32 (9.4)	0/5	3/34 (8.8)	4/28 (14.3)	7/52 (13.5)
Thrombocytopenia	23/199 (11.6)	2/32 (6.2)	0/5	2/34 (5.9)	2/28 (7.1)	3/52 (5.8)
**SDMA**						
Increased SDMA	64/197 (32.5)	9/35 (25.7)	2/5 (40)	10/38 (26.3)	13/26 (50)	19/54 (35.2)
**sCr**						
Increased sCr	18/205 (8.8)	2/36 (5.6)	1/5 (20)	2/38 (5.3)	5/27 (18.5)	6/55 (10.9)
**BUN**						
Increased BUN	27/177 (15.2)	5/33 (15.2)	2/4 (50)	5/34 (14.7)	6/23 (26.1)	10/50 (20)
Decreased BUN	12/177 (6.8)	2/33 (6.1)	2/4 (50)	2/34 (5.9)	1/23 (4.3)	3/50 (6)
**PHOS**						
Increased PHOS	47/140 (33.6)	5/22 (22.7)	0/2	5/23 (21.7)	2/18 (11.1)	7/38 (18.4)
**USG**						
USG < 1035	56/208 (26.9)	11/36 (30.6)	3/4 (75)	12/37 (32.4)	8/26 (30.8)	17/54 (31.5)
**UPC**						
BP	19/159 (11.9)	3/30 (10)	0/4	3/31 (9.7)	2/16 (12.5)	3/39 (7.7)
P	21/159 (13.2)	5/30 (16.7)	2/4 (50)	5/31 (16.1)	3/16 (18.7)	6/39 (15.3)
**tT4**						
Increased tT4	10/38 (26.3)	1/11 (9.1)	0/1	1/12 (8.3)	2/7 (28.6)	3/14 (21.4)

**Table 5:** Description of significant (*) Spearman’s rank correlation coefficient (*r_s_*) and *p* values of cat complete blood count (CBC) and renal profile abnormalities according to their FeMV positivity by qPCR_FeMV_ from urine (u^+^), blood (b^+^), urine and/or blood (u^+^ and/or b^+^), FeMV antibodies (Ab^+^), FeMV antibodies and/or qPCR_FeMV_ (Ab^+^ and/or RNA^+^). sCr = serum creatinine; USG = urine specific gravity.

Variable	u^+^	b^+^	u^+^ and/or b^+^	Ab^+^	Ab^+^ and/or RNA^+^
Anemia					
*r_s_*	0.075	0.006	0.063	0.136	0.172
*p*	0.265	0.930	0.368	0.060	0.020 *
Neutrophilia					
*r_s_*	0.163	0.080	0.164	0.191	0.274
*p*	0.023 *	0.271	0.025 *	0.012 *	0.000 *
Monocytosis					
*r_s_*	0.132	0.266	0.198	0.060	0.207
*p*	0.067	0.000 *	0.007 *	0.429	0.007 *
Eosinopenia					
*r_s_*	−0.050	−0.151	−0.090	0.069	−0.032
*p*	0.485	0.037 *	0.222	0.367	0.682
Basophilia					
*r_s_*	0.165	−0.12	0.158	0.174	0.117
*p*	0.021 *	0.870	0.031 *	0.022 *	0.130
Thrombocytosis					
*r_s_*	0.126	0.037	0.129	0.160	0.249
*p*	0.077	0.610	0.075	0.032 *	0.001 *
sCr					
*r_s_*	−0.053	0.071	−0.044	0.145	0.077
*p*	0.454	0.319	0.539	0.049 *	0.308
USG					
*r_s_*	−0.037	−0.165	−0.084	−0.038	−0.087
*p*	0.591	0.020 *	0.240	0.609	0.247

**Table 6:** Number of cats in each clinical group (*n*) positive to FeMV according to the positivity by qPCR_FeMV_ from urine (u^+^), blood (b^+^), urine and/or blood (u^+^ and/or b^+^), FeMV antibodies (Ab^+^), FeMV Abs and/or qPCR_FeMV_ (Ab^+^ and/or RNA^+^). CKD = chronic kidney disease.

Clinical Group	*n*	u^+^	b^+^	u^+^ and/or b^+^	Ab^+^	Ab^+^ and/or RNA^+^
Healthy	12	1	0	1	1	2
Unhealthy without CKD	92	20	1	20	8	23
CKD	88	15	3	16	15	27

**Table 7:** Prevalence of the other investigated viruses with description of number (*n*) of serum and EDTA blood samples tested for antibody detection and PCR respectively for each virus.

Virus and Sample	*n*	Positive (%)
FIV		
Serum	209	27 (12.9)
EDTA blood	211	21 (10)
FeLV		
EDTA blood	211	3 (1.4)
FCoV		
EDTA blood	174	33 (19)
FPV		
EDTA blood	105	8 (7.6)

**Table 8:** Description of significant (*) Spearman’s rank correlation coefficient (*r_s_*) and *p* values of variables significantly correlated to coinfection between FeMV (Ab^+^ and/or RNA^+^) and FIV (Ab^+^ and/or RNA^+^) or FeMV (Ab^+^ and/or RNA^+^) and FCoV (RNA^+^) when compared to cats that were positive only for FeMV (Ab^+^ and/or RNA^+^).

Variable	FeMV and FIV	FeMV and FCoV
Age		
*r_s_*	0.396	0.000
*p*	0.003 *	1.000
Enlarged Lymph nodes		
*r_s_*	0.412	−0.065
*p*	0.002 *	0.651
BCS		
*r_s_*	−0.331	−0.239
*p*	0.013 *	0.095
MCS		
*r_s_*	−0.161	−0.324
*p*	0.251	0.026 *
Thrombocytosis		
*r_s_*	0.011	0.329
*p*	0.936	0.024 *

**Table 9:** List of the four infection patterns obtained testing with molecular (qPCR_FeMV_ u and/or b) and serological (Ab) assays related to FeMV and number (*n*) of cats showing any single pattern.

qPCR_FeMV_ u and/or b	Ab	*n*
Positive	Positive	8
Positive	Negative	28
Negative	Positive	20
Negative	Negative	128

**Table 10:** Description of significant associations detected by Fisher’s exact test of the three infection patterns obtained with molecular (qPCR_FeMV_ u and/or b) and serological (Ab) investigations related to FeMV with the investigated variables when compared to qPCR_FeMV_ u and/or b and Ab negative pattern. Results are represented by *p* values, OR (odds ratio) and 95% CI (confidence intervals).

qPCR_FeMV_ u and/or b	Ab	Variable	*p*	OR	95% CI
Positive	Positive	Oral lesions	0.0377	5.095	1.371–18.32
		FIV RNA^+^	0.0134	10.37	2.286–57.91
Positive	Negative	Single-cat household	0.017	11.53	1.672–133.6
		Neutrophilia	0.04	2.949	1.048–8.073
		Monocytosis	0.007	4.058	1.552–11.15
Negative	Positive	Low BCS	0.0071	4.353	148–11.19
		Oral lesions	0.0288	3.397	1.96–9.656
		Gastrointestinal signs	0.0303	5.083	1.467–17.52
		Anemia	0.0094	4.074	1.399–10.56
		Neutrophilia	0.0048	4.524	1.651–11.74

**Table 11:** Indirect immunofluorescence (IIF) and PCR (blood and/or urine) test results in FeMV positive followed up cats. Results refer to the sampling day. d = day; pos = positive; neg = negative.

	0	3 d	7 d	10 d	14 d	21 d	30 d	45 d	60 d	75 d	90 d	120 d	150 d	180 d	210 d	240 d	270 d	300 d	360 d	510 d	570 d
Cat 1.																					
IIF assay																					
EDTA blood PCR	neg																				
Urine PCR	pos								pos												
Cat 2.																					
IIF assay	pos																	neg			
EDTA blood PCR	neg																	neg	neg		neg
Urine PCR	pos												neg						pos		neg
Cat 3.																					
IIF assay	neg						neg											neg			
EDTA blood PCR	neg						neg											neg			
Urine PCR	pos						pos								pos	pos		neg			
Cat. 4																					
IIF assay	neg					pos															
EDTA blood PCR	pos					pos															
Urine PCR	neg																				
Cat 5.																					
IIF assay	neg																		neg		
EDTA blood PCR	neg																				
Urine PCR	pos																		pos		
Cat 6.																					
IIF assay						pos															
EDTA blood PCR						neg								neg							
Urine PCR	pos					pos															
Cat 7.																					
IIF assay	pos						pos														
EDTA blood PCR	neg						neg														
Urine PCR	neg						neg														
Cat 8.																					
IIF assay	neg																neg				neg
EDTA blood PCR	neg																pos	pos			neg
Urine PCR	neg		neg		neg		neg	neg	neg		neg	neg									neg
Cat 9.																					
IIF assay	neg					neg															
EDTA blood PCR	neg																				
Urine PCR	pos					pos	pos														
Cat 10.																					
IIF assay	pos	pos								neg											
EDTA blood PCR	neg	neg								neg			neg								
Urine PCR	neg	pos		pos	pos		pos	pos	pos	neg		neg	neg	neg							
Cat 11.																					
IIF assay	pos																			neg	
EDTA blood PCR	neg																			neg	
Urine PCR	neg																			neg	
Cat 12.																					
IIF assay	pos																				
EDTA blood PCR	neg								neg												
Urine PCR	pos								pos												
Cat 13.																					
IIF assay	neg																				
EDTA blood PCR	neg																				
Urine PCR	pos								pos		pos										

## Data Availability

The data set analyzed for the current study is available from the corresponding author upon reasonable request.
